# The search for optimal nocturnal diurnal heart rate Index targets in ICU patients: a retrospective observational study from large ICU database

**DOI:** 10.3389/fcvm.2024.1415467

**Published:** 2024-07-08

**Authors:** Lan Gao, QinDong Shi, XiaoYu Zhang, Xiang Bu, PeiYing Zheng, LinJing Zhou, JinQi Yan, Hao Li, Gang Tian

**Affiliations:** ^1^ Department of Critical Care Medicine, The First Affiliated Hospital of Xi’an Jiaotong University, Xi’an, Shaanxi, China; ^2^ Shaanxi Provincial Key Laboratory of Sepsis in Critical Care Medicine, Xi’an Jiaotong University, Xi’an, Shaanxi, China; ^3^ Department of Cardiology, The First Affiliated Hospital of Xi’an Jiaotong University, Xi’an, Shaanxi, China

**Keywords:** nocturnal diurnal heart rate index (NDHRI), circadian rhythms, ICU patients, mortality, optimal target range, eICU collaborative research database

## Abstract

**Background:**

Circadian rhythms play a crucial role in cardiovascular health, with the nocturnal diurnal heart rate index (NDHRI) reflecting significant circadian variations. However, the optimal NDHRI target in Intensive Care Unit (ICU) patients remains undefined. This study aims to establish an evidence-based NDHRI target range and assess its association with mortality.

**Methods:**

Data from the eICU Collaborative Research Database (*n* = 32,412) were analyzed. NDHRI was calculated by dividing cumulative nighttime heart rate area by daytime area. Generalized additive models (GAMs) explored the non-linear relationship between mean NDHRI and mortality, adjusting for confounders. Subgroup analyses were conducted based on ethnicity, ICU type, and comorbidities.

**Results:**

A U-shaped association was observed between hospital mortality and mean NDHRI (*P* < 0.001). The optimal NDHRI range (40.0%–45.0%) demonstrated the lowest mortality rates. The duration spent within this range correlated inversely with mortality (*P* < 0.001). Subgroup analyses consistently supported these findings across diverse patient profiles.

**Conclusions:**

Our findings suggest an association between maintaining NDHRI within the 40.0%–45.0% range and lower mortality rates in critically ill patients, highlighting the potential utility of monitoring circadian heart rate variations in the ICU. Further research and future randomized controlled trials are essential to confirm causality and should consider this NDHRI range as a pivotal reference target.

## Key points

**Critical Importance of NDHRI:** Our study underscores the critical role of the Nocturnal to Diurnal Heart Rate Index (NDHRI) in predicting hospital and ICU mortality in critically ill patients, revealing a U-shaped relationship.

**Optimal NDHRI Range:** We propose a targeted NDHRI range of 40.0%–45.0%, demonstrating the lowest mortality rates. This range holds broad applicability across diverse ethnicity, ICU type, and comorbidities.

**Proactive Monitoring:** NDHRI offers a valuable tool for early diagnosis, continuous monitoring, and personalized treatment strategies, enhancing the proactive management of critically ill patients in the Intensive Care Unit (ICU).

## Introduction

Circadian rhythms represent a ubiquitous and robust biological phenomenon across living organisms. Similar to blood pressure, human heart rate displays a prominent circadian rhythmicity characterized by a significant nocturnal reduction, followed by an increase upon morning awakening (1, 2). Crucially, this circadian heart rate variation intricately links with normal physiological processes (3, 4), exerting substantial influence on circadian cardiac output and myocardial contractility while closely associating with the timing of cardiovascular events (5–7).

A series of multicenter prospective clinical studies, encompassing research conducted in China and various regions, has yielded critical insights: (1) The 24-h average heart rate can predict overall mortality and non-cardiovascular disease-related mortality but lacks effectiveness in predicting mortality associated with cardiovascular diseases; (2) Daytime heart rate proves insufficient in predicting overall mortality or any fatal or non-fatal events; (3) Nocturnal heart rate serves as an indicator of overall mortality but lacks predictive power for fatal or non-fatal events; (4) The night-to-day heart rate ratio exhibits the potential to predict overall mortality, mortality unrelated to cardiovascular diseases, and other fatal or non-fatal events beyond stroke (8). Furthermore, research conducted by Japanese scholars, including Hozawa et al., has established a significant correlation between the night-to-day heart rate ratio and long-term overall mortality in non-cardiovascular patients (1). Additionally, investigations focused on hypertensive patients have revealed a noteworthy association between an elevated heart rate during nighttime sleep and an increased risk of new cardiovascular events and overall mortality, independent of daytime wakefulness (9). Patients with a high night-to-day heart rate ratio face a significantly elevated risk of cardiovascular events (10). Collectively, these studies underscore the enhanced predictive value of the night-to-day heart rate ratio for mortality and the incidence of adverse events.

It is imperative to acknowledge that the relationship between the night-to-day heart rate ratio and clinical outcomes may not adhere to a linear pattern. Patients with a low night-to-day heart rate ratio, indicating a relatively slow nocturnal heart rate, may lead to inadequate organ perfusion, potentially triggering cardiovascular malignant events and cerebral hypoperfusion, resulting in syncope or coma (11–13). Conversely, a high night-to-day heart rate ratio, indicating a relatively rapid nocturnal heart rate, may increase cardiac burden, ultimately elevating the long-term risks of conditions such as hypertension, myocardial infarction, and heart failure, accompanied by discomfort, anxiety, and exacerbation of underlying medical conditions (14–16). Therefore, we postulate that the relationship between the night-to-day heart rate ratio and clinical outcomes may assume a U-shaped pattern, although current empirical studies offer limited direct support for this hypothesis.

However, within the context of Intensive Care Unit (ICU) patients, the optimal range for controlling the night-to-day heart rate ratio remains undefined, and practical guidance concerning targets for night-to-day heart rate control is conspicuously absent. A critical challenge arises from the lack of scientific evidence to substantiate the determination of the optimal night-to-day heart rate ratio control target. Consequently, there is an urgent need for large-scale multicenter research aimed at delineating the optimal target range for the night-to-day heart rate ratio, thereby providing robust guidance for clinical practice and future research endeavors.

In the current landscape, we are well-positioned to harness the potential of substantial data resources, including accessible de-identified datasets such as the eICU Collaborative Research Database (eICU-CRD) (17), which contains comprehensive information regarding patients admitted to the Intensive Care Unit. Our research endeavors avoid reliance on linear assumptions, with the primary objective being the establishment of the optimal range for night-to-day heart rate control targets by correlating the night-to-day heart rate ratio with mortality. Subsequently, we shall undertake a comprehensive assessment of the relationship between the duration spent within this optimal range and mortality rates. The outcomes of this study are anticipated to provide scientifically grounded recommendations for heart rate management in ICU patients, thereby enhancing the quality of clinical healthcare management and offering high-quality data support for future research initiatives.

## Methods

### Data source

We obtained our data from the eICU Collaborative Research Database v2.0. This extensive public database was created through a collaboration between Philips Healthcare and the Laboratory for Computational Physiology (LCP) at the Massachusetts Institute of Technology (MIT). It encompasses routine data from 200,859 patients admitted to intensive care units (ICUs) in 208 hospitals across the United States during the years 2014 and 2015. The database contains a wealth of high-quality clinical information, including vital signs, demographic records, nursing plans, disease severity assessments, diagnostic data, and treatment details (18).

Data collection adhered to ethical standards outlined by the Institutional Review Board of the Massachusetts Institute of Technology (IRB protocol no. 0403000206) and the principles of the 1964 Declaration of Helsinki, including its later amendments. Access to the database was granted to the author with certification number 33365315, and the responsibility for data extraction lay with the author. This study follows the reporting guidelines outlined in the STrengthening the Reporting of Observational Studies in Epidemiology (STROBE) statement (19).

### Study focus: outcomes and key variable

The primary outcome of interest in this study was hospital mortality, with ICU mortality considered as a secondary outcome. The primary independent variable under investigation was the nocturnal diurnal heart rate index (NDHRI), irrespective of whether interventions to manage heart rate were implemented.

### Calculation of NDHRI

**Figure 1:**
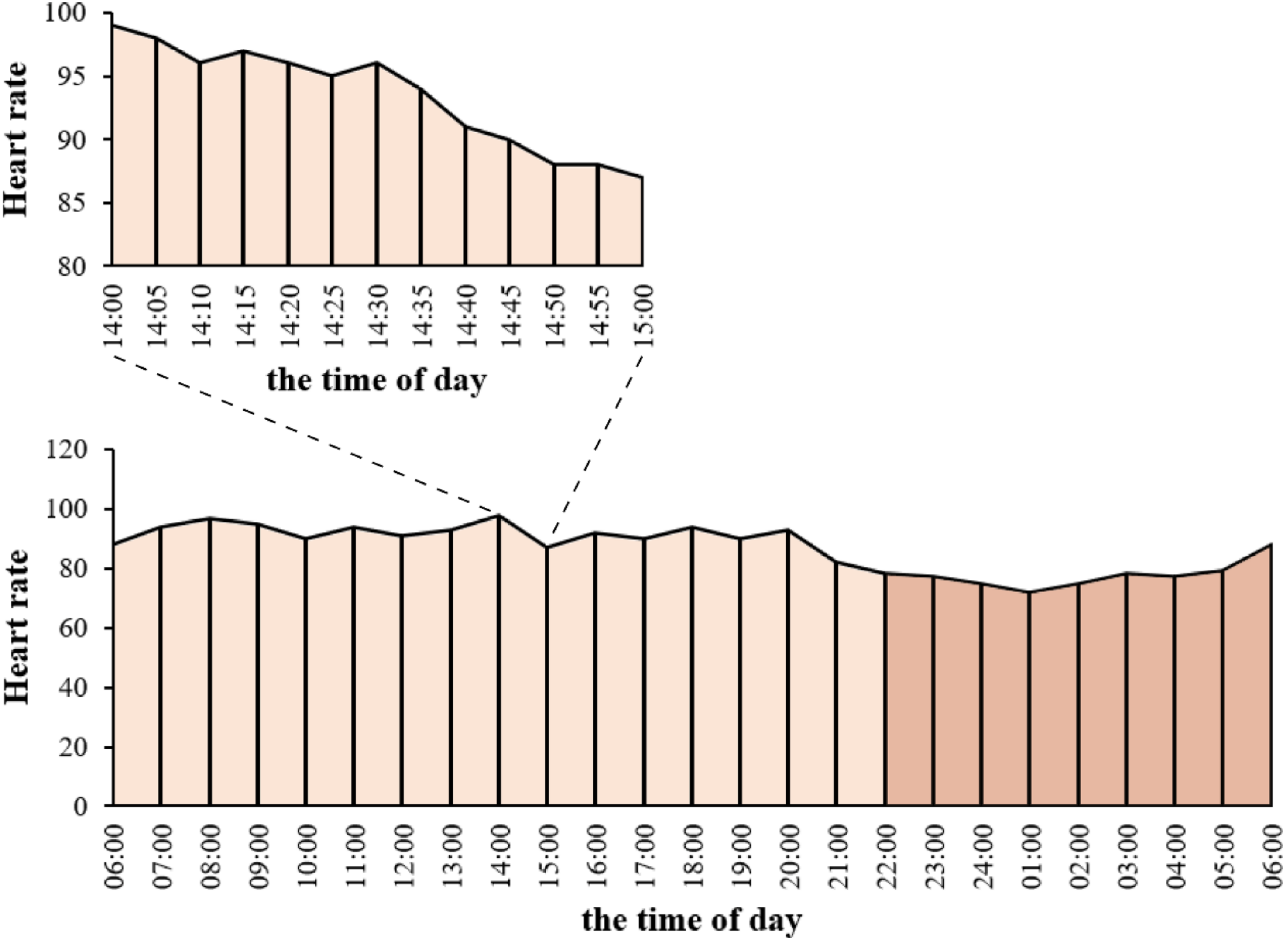
NDHRI calculation. Heart rate measurements from the eICU-CRD dataset were recorded every 5 min as bars, with width indicating time and height corresponding to heart rate. Bar area was calculated as the product of 5 min and heart rate. Nighttime (22:00–6:00) cumulative area (deep orange) and daytime (6:00–22:00) cumulative area (light orange) were determined by summing bar areas within their respective periods. NDHRI is the ratio of nighttime to daytime cumulative areas.

The division of heart rates into day and night periods was defined as follows: Nighttime spanned from 22:00 in the evening to 6:00 the following morning, while daytime extended from 6:00 in the morning to 22:00 in the evening. Heart rate measurements were recorded consecutively every 5 min within the eICU-CRD dataset. Each recorded heart rate was treated as the average for the corresponding 5-min interval, resulting in the creation of a timeline represented by bars. The base width of each bar corresponded to 5 min, and the height was proportional to the recorded heart rate value. Consequently, the area of each bar was calculated by multiplying 5 min by the heart rate value. For the nighttime period (from 22:00 to 6:00), the cumulative area under the heart rate timeline was computed by summing the areas of all bars within that timeframe. Similarly, for the daytime period (from 6:00 to 22:00), the cumulative area under the heart rate timeline was determined by summing the areas of all bars within that interval. The ratio of the nighttime cumulative area to the daytime cumulative area on the heart rate timeline was designated as the nocturnal diurnal heart rate index (NDHRI) (Figure 1). In a novel approach to assessing circadian rhythms, this method provides a distinct measure of circadian rhythms by utilizing the ratio of nighttime to daytime cumulative heart rate areas, allowing for an analysis of broader variations across ICU stays and offering a departure from conventional point-by-point analysis (20).

For each patient, the NDHRI could be calculated daily. The mean NDHRI was obtained by summing the NDHRI values for each day of their ICU stay and dividing by the total number of days spent in the ICU. The mean NDHRI during a patient's hospitalization was used as an indicator to assess the central tendency of heart rate management. Additionally, the proportion of NDHRI measurements falling within a specified range was considered to evaluate heart rate management.

### Patient selection

In this study, we considered all patient admissions from the eICU-CRD database as potential candidates for inclusion. To maintain data integrity and align with our research objectives, we established specific exclusion criteria, which encompassed: (1) Cases with missing consecutively recorded heart rate data. From our initial dataset of 200,859 patients, we had to exclude 115,912 patients due to missing consecutively recorded heart rate data. We performed tests to assess the randomness of these missing data, which indicated that the missingness was not completely random. This non-randomness may be associated with specific hospital recording systems or data collection biases during certain periods. (2) Instances of multiple ICU stays by the same patient, including multiple ICU admissions during a single hospital stay, and multiple ICU stays throughout the entire study period. (3) Missing or right-censored age information. (4) Patients under the age of 18. (5) Absence of recorded gender. (6) Unavailability of body mass index (BMI) data. (7) Patients discharged or deceased within a single calendar day from their initial hospitalization. (8) Heart rate records with values significantly deviating from the norm, defined as exceeding the mean by more than three times the standard deviation. (9) Patients with a documented history of cardiac arrhythmias, cardiac arrest, or those who use a cardiac pacemaker for any underlying medical reasons. (10) Patients with incomplete data regarding hospital or ICU mortality outcomes. Adhering to these exclusion criteria was crucial to ensure the quality and relevance of the patient cohort included in our analysis.

### Data collection

We extracted the following variables from the eICU-CRD database: demographic parameters (including age, gender, BMI, and ethnicity), types of ICUs (Medical-Surgical ICU, Cardiac ICU, Coronary Care Unit-Cardiothoracic ICU, Cardiothoracic Surgery ICU, Cardiothoracic ICU, Medical ICU, Neurological ICU, Surgical ICU), comorbidity parameters [such as hypertension, coronary artery disease, heart failure, chronic obstructive pulmonary disease (COPD), diabetes mellitus, chronic liver disease, upper gastrointestinal bleeding, chronic renal insufficiency, stroke, cancer, and bone fractures], Acute Physiology and Chronic Health Evaluation (APACHE) IV score, duration of ICU stay, duration of in-hospital stay, ICU mortality, in-hospital mortality, and heart rate measurements (recorded consecutively every 5 min).

### Statistical analysis

**Figure 2:**
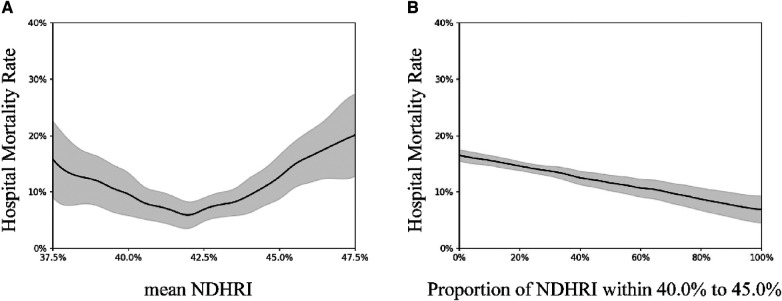
These visual summaries are based on generalized additive model analyses conducted on a dataset comprising 32,412 ICU admissions from the eICU-CRD dataset. (**A**) Illustrates the correlation between hospital mortality and mean NDHRI, while (**B**) presents the relationship between hospital mortality and the proportion of time spent within the 40%–45% NDHRI range. The black solid line represents the average prediction, and the gray shaded area indicates the 95% confidence interval.

The observed associations between elevated and diminished night-to-day heart rate ratios and adverse outcomes (21, 22) have provided compelling evidence supporting a non-linear correlation between NDHRI and mortality. To address this non-linearity, we utilized Generalized Additive Models (GAMs) (23), a well-established method widely accepted in scientific research for conducting multivariable regression analysis (24). In this study, we specifically employed a log link function in the GAM to handle ratio data and improve the predictive accuracy of the model. These GAMs enabled us to precisely estimate the relationship between mean NDHRI and mortality while meticulously controlling for potential confounding variables, including age, gender, BMI, and the APACHE IV score recorded on the initial day of ICU admission. The APACHE IV score, based on data collected within the first 24 h of ICU admission, provides a valuable estimate of patient mortality (25, 26). Furthermore, considering the availability of the APACHE IV score in the eICU-CRD dataset, we used this score in lieu of the SOFA score as a control variable. In our analysis of the eICU-CRD dataset, we introduced a random hospital intercept to account for correlations among cases originating from the same medical facility, effectively mitigating biases associated with inter-hospital variations. During these analyses, a significant U-shaped relationship between mean NDHRI and hospital mortality was evident (*P* < 0.001). Given the clinical significance of this finding, we conducted a thorough post-hoc analysis to further investigate and define the specific NDHRI range associated with the lowest mortality risk. This targeted analysis, supported by visual evidence in Figure 2A, is detailed in the results section and played a crucial role in establishing optimal NDHRI thresholds. The results of these analyses were then applied to specify the optimal NDHRI range. Following this determination, we conducted a comprehensive assessment of the association between mortality and the proportion of NDHRI values falling within this specified range. We systematically considered the possibility of non-linear associations for all continuous predictors. Additionally, subgroup analyses were conducted, including stratification by ethnicity, ICU type, and the presence of comorbidities such as hypertension, heart failure, and chronic obstructive pulmonary disease (COPD).

## Results

**Figure 3:**
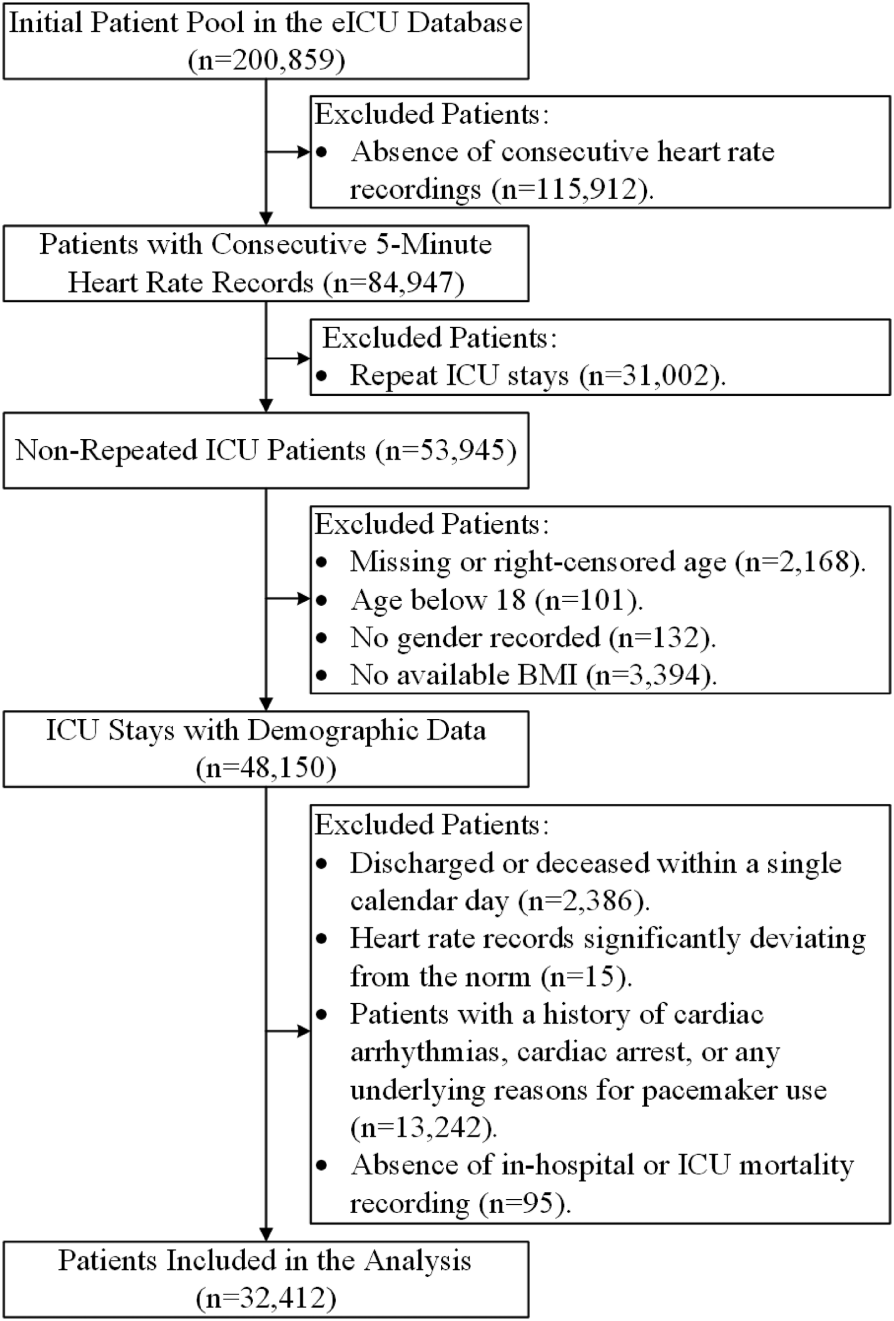
Flowchart of patient inclusion. This visual representation illustrates the selection process that led to the inclusion of 32,412 ICU stays for analysis from the initial pool of 200,859 ICU stays in the eICU-CRD dataset.

Figure 3 outlines the meticulous process of selecting 32,412 ICU stays meeting our inclusion criteria from the extensive pool of 200,859 ICU stays available in the eICU-CRD dataset for our comprehensive analysis. Table 1 provides a comprehensive summary of the demographic and clinical characteristics of this selected cohort.

**Table 1:** Demographic and clinical characteristics.

Variables	Data (*n* = 32,412)
Age (year)	62.6 ± 16.4
Male, *n* (%)	17,795 (54.9%)
BMI (kg/m^2^)	29.3 ± 7.9
Ethnicity, *n* (%)	
Caucasian	24,179 (74.6%)
African American	3,825 (11.8%)
Hispanic	1,685 (5.2%)
Asian	681 (2.1%)
Native American	259 (0.8%)
Other/unknown	1,783 (5.5%)
ICU types, *n* (%)	
Med-Surg ICU	17,178 (53.0%)
Cardiac ICU	2,593 (8.0%)
CCU-CTICU	2,917 (9.0%)
CSICU	972 (3.0%)
CTICU	973 (3.0%)
MICU	2,917 (9.0%)
Neuro ICU	2,593 (8.0%)
SICU	2,269 (7.0%)
Comorbidities, *n* (%)	
Hypertension	17,114 (52.8%)
Coronary artery disease	3,987 (12.3%)
Heart failure	4,797 (14.8%)
COPD	2,885 (8.9%)
Diabetes mellitus	8,622 (26.6%)
Chronic liver disease	1,977 (6.1%)
Upper gastrointestinal bleed	745 (2.3%)
Chronic renal insufficiency	3,338 (10.3%)
Stroke	2,755 (8.5%)
Cancer	4,019 (12.4%)
Bone fractures	551 (1.7%)
APACHE Ⅳ score [median (IQR)]	54 [40, 71]
ICU length of stay, d [median (IQR)]	4 [3, 5]
Number of recorded nights in ICU [median (IQR)]	
Survivors	4 [3, 5]
Non-survivors	4 [3, 6]
Total	4 [3, 5]
Total number of recorded nights in ICU	
Survivors	1,14,480
Non-survivors	15,168
Total	1,29,648
Hospital length of stay, d [median (IQR)]	6 [3, 9]
ICU mortality, *n* (%)	2,042 (6.3%)
Hospital mortality, *n* (%)	3,792 (11.7%)
Mean NDHRI	43.2% ± 5.6%
Mean prop. of NDHRI within 40.0%–45.0%	42.9% ± 9.8%

The data is presented as mean ± standard deviation, median [interquartile range], or number (percentages). IQR, interquartile range; BMI, body mass index; ICU, intensive care unit; Med-Surg ICU, medical-surgical ICU; CCU-CTICU, coronary care unit-cardiothoracic ICU; CSICU, cardiothoracic surgery ICU; CTICU, cardiothoracic ICU; MICU, medical ICU; neuro ICU, Neurological ICU; SICU, surgical ICU; APACHE, acute physiology and chronic health evaluation; NDHRI, nocturnal diurnal heart rate index. Number of recorded nights in ICU refers to the median number of nights during which patient heart rate data was recorded. Total number of recorded nights in ICU is the sum of all recorded nights for survivors and non-survivors, indicating the total amount of data collected.

Figure 2A illuminates a compelling U-shaped relationship between hospital mortality and mean NDHRI (*P* < 0.001), underscoring the impact of both high and low NDHRI values on an increased risk of hospital mortality. Recognizing this noteworthy association, we felt compelled to define a specific NDHRI range, encompassing both lower and upper thresholds. Through a detailed *post-hoc* analysis, our inspiration for this range was derived from the most smoothly varying portion of the U-shaped curve depicted in Figure 2A, leading to the delineation of an NDHRI range spanning from 40.0% to 45.0%. Table 2 corroborates this finding, with mean NDHRI values selected as 40.0%, 42.5%, and 45.0%, taking into account the nadir of the U-shaped curve in Figure 2A, approximately at 42.5%, and ensuring symmetry by including values at 40.0% and 45.0%. Importantly, Table 2 consistently reveals that both high and low NDHRI values are associated with an increased risk of hospital mortality, irrespective of adjustments made for potential confounders.

**Table 2:** Odds ratios of hospital mortality (with 95% confidence intervals) from generalized additive models: impact of mean NDHRI measurements and the defined NDHRI range.

** **	NDHRI			
	40.0% vs. 42.5%	*P* value	45.0% vs. 42.5%	*P* value
Unadjusted	4.31 (1.66–11.23)	0.003	6.73 (2.81–16.11)	<0.001
Adjusted	3.36 (1.33–8.46)	0.009	4.41 (1.76–11.08)	0.002

Odds ratios for hospital mortality, with 95% confidence intervals in parentheses, were computed through generalized additive models (GAMs). The *P*-values reported for the comparison of NDHRI measurements (40.0% vs. 42.5% and 45.0% vs. 42.5%) are derived from Wald tests associated with the model coefficients in the GAMs. These analyses were performed in both unadjusted and adjusted settings, controlling for confounders such as age, gender, BMI, and the APACHE IV score recorded on the initial day of ICU admission. The Wald test evaluates the null hypothesis that the coefficient of an NDHRI measurement in the model is zero (indicating no effect) against the alternative hypothesis that the coefficient is not zero (indicating a significant effect).

Subsequently, we aimed to explore the relationship between the duration spent within this defined NDHRI range and hospital mortality. Figure 2B aptly illustrates the strong correlation between hospital mortality rates and the time spent within this specified range. Notably, these associations remain robust and highly significant in the eICU-CRD dataset, with a *p*-value of less than 0.001.

**Figure 4:**
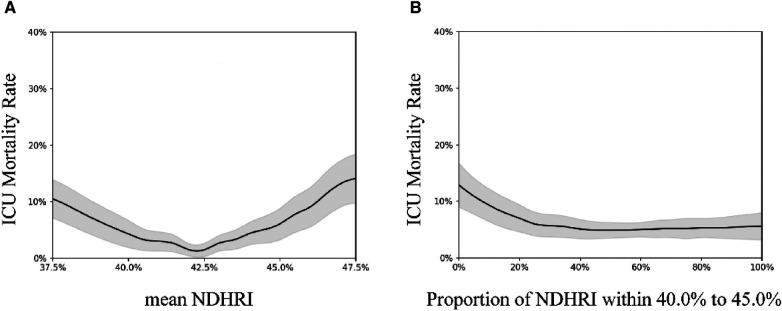
These figures illustrate the correlation between ICU mortality and mean NDHRI (**A**) and explore the connection between ICU mortality and the proportion of time spent within the 40.0%–45.0% NDHRI range (**B**) likewise, (**A**) demonstrates a U-shaped association, while (**B**) exhibits a declining trend.

Furthermore, as evident in both Figures 4A,B, a statistically significant U-shaped association between ICU mortality and mean NDHRI emerges, along with a declining trend between ICU mortality and the proportion of time spent within the 40.0%–45.0% NDHRI range, both with *p*-values less than 0.001.

**Figure 5:**
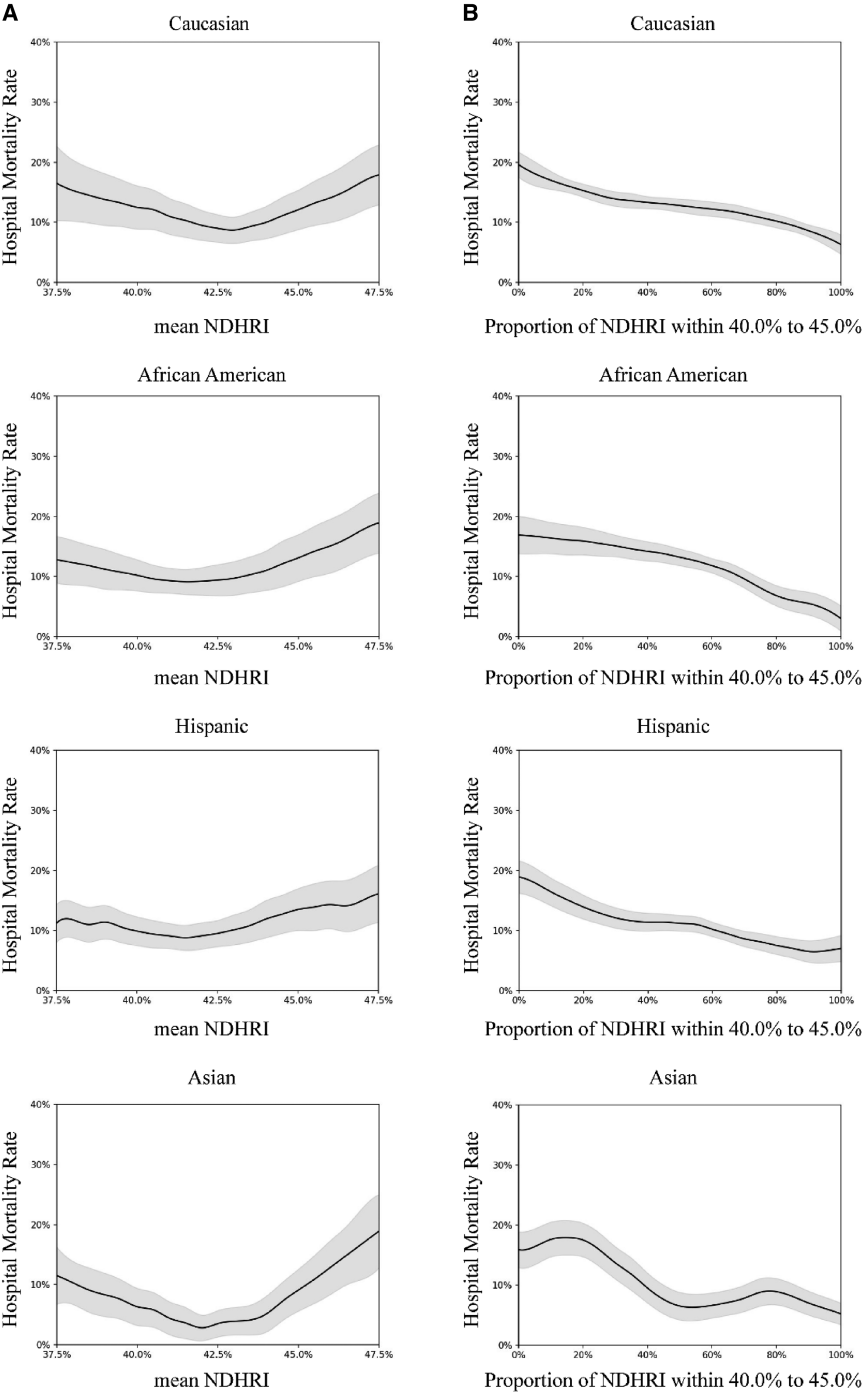
Subgroup analysis by ethnicity. In this subgroup analysis, we explored the impact of different ethnicities. The results were drawn from data in the eICU-CRD dataset, encompassing 24,179 Caucasians, 3,825 African Americans, 1,685 Hispanics, and 681 Asians. (**A**) Portrays the connection between hospital mortality and the mean NDHRI, categorized by patient ethnicity. These plots closely resemble Figure 2A in the main text. (**B**) Parallels Figure 2B in the main text, illustrating the relationship between hospital mortality and the proportion of time spent with an NDHRI falling within the range of 40.0%–45.0%, categorized by patient ethnicity. These subgroup analysis findings shed light on the disparities in hospital mortality, mean NDHRI levels, and the time spent within specific NDHRI ranges among patients of various ethnic backgrounds.

**Figure 6:**
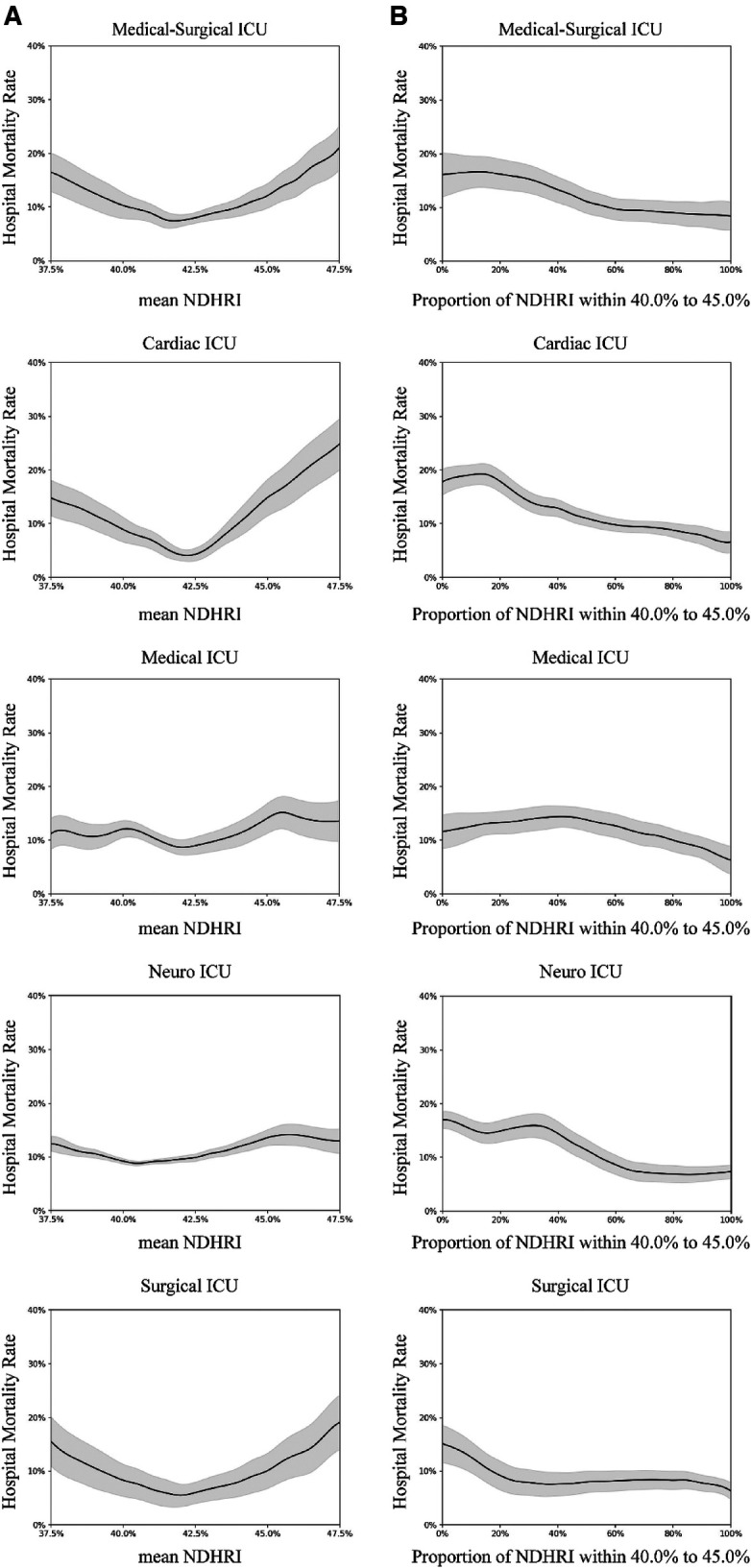
Subgroup analysis by intensive care unit (ICU) type. In this subgroup analysis, we explored various types of intensive care units (ICUs) using data from the eICU-CRD dataset. The dataset included hospital admissions from different ICU categories: 17,178 stays in Medical-Surgical ICUs, 2,593 stays in Cardiac ICUs, 2,917 stays in Medical ICUs, 2,593 stays in Neuro ICUs, and 2,269 stays in Surgical ICUs. (**A**) Illustrates the correlation between hospital mortality and the mean NDHRI, stratified by ICU type. This analysis aligns with the methodology presented in Figure 2A in the main text. (**B**) Closely resembles Figure 2B in the main text, presenting the relationship between hospital mortality and the proportion of time spent with NDHRI falling within the range of 40.0%–45.0%, categorized by ICU type. These subgroup analysis findings contribute significantly to our understanding of disease outcomes and NDHRI levels across diverse ICU types.

**Figure 7:**
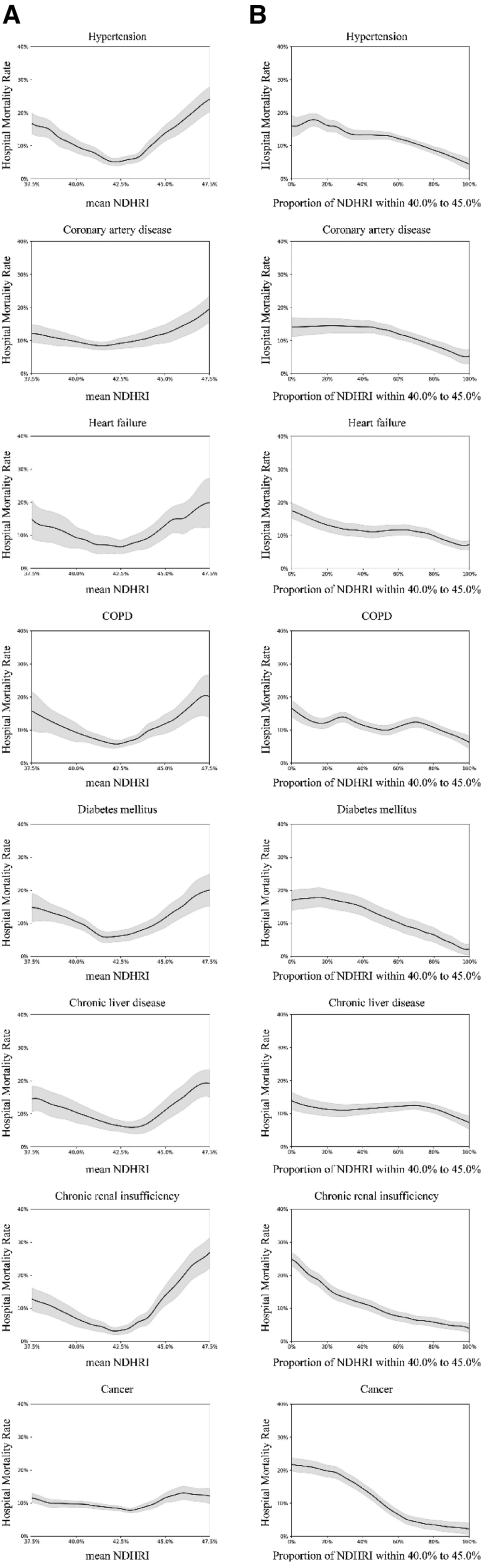
Subgroup analysis on diagnoses and comorbidities: using the eICU-CRD dataset, this analysis explores diagnoses identified by ICD-9 codes, covering hypertension (17,114 patients), coronary artery disease (3,987 patients), heart failure (4,797 patients), chronic obstructive pulmonary disease (COPD) (2,885 patients), diabetes mellitus (8,622 patients), chronic liver disease (1,977 patients), chronic renal insufficiency (3,338 patients), and cancer (4,019 patients). (**A**) Examines the correlation between hospital mortality and mean NDHRI in the context of diagnoses and comorbidities. Simultaneously, (**B**) illustrates the relationship between hospital mortality and the proportion of time spent with NDHRI in the 40.0%–45.0% range, considering diagnoses and comorbidities. (**A**) Shows a U-shaped relationship, while (**B**) consistently indicates a downward trend across various diagnostic and comorbidity subgroups.

Subgroup analyses were conducted, including stratification by ethnicity, ICU type, and the presence of comorbidities. These analyses are detailed in Figures 5–7.

## Discussion

Our in-depth examination of the eICU-CRD database has disclosed a pronounced U-shaped relationship between hospital mortality and the Nocturnal Diurnal Heart Rate Index (NDHRI) among patients in the Intensive Care Unit (ICU). Notably, the lowest rates of mortality were observed when the average NDHRI fell within the range of 40.0%–45.0%, particularly when patients spent a substantial amount of time within this defined range. Although some subgroups displayed increased variability due to limited sample sizes, the consistent patterns observed across various subgroup analyses, including different ethnic groups, ICU types, and diagnostic comorbidity categories, affirm the robustness of our findings. This consistency was evident even when using ICU ward mortality as a proxy for hospital mortality, demonstrating the reliability of our conclusions across diverse patient demographics and clinical settings.

Regarding the substantial data missingness in the initial dataset from the eICU-CRD database due to the lack of consecutively recorded heart rate data, we recognize that this may impact the generalizability of our study results. To assess this impact, we conducted sensitivity analyses comparing models with and without the excluded data. The results indicated that, despite the exclusion of a large amount of data, the stability and directionality of the research conclusions remained consistent across models. This suggests that our findings are robust, although we must still cautiously interpret the potential biases introduced by data missingness.

It is important to acknowledge that individual sleep patterns of ICU patients might differ from conventional circadian rhythms (27). Yet, our choice of day and night segments was informed by the typical rhythms observed in hospital settings. This empirical approach provided a stable framework for analyzing heart rate variations, taking into account the unique pathophysiological states of ICU patients (28).

The observed U-shaped relationship between NDHRI and hospital mortality in this study profoundly reflects the impact of heart rate variations on physiological states, showing that a very low NDHRI might lead to slow nocturnal heart rates, affecting vital organ perfusion, while a very high NDHRI might reflect excessive stress responses at night, increasing cardiac load. For instance, in the subgroup analysis of comorbidities, patients with hypertension, coronary artery disease, and heart failure exhibited a significant U-shaped curve. In hypertensive patients, a high NDHRI may reflect persistent high sympathetic activity at night, increasing the risk of cardiovascular events; a very low NDHRI might indicate insufficient nocturnal blood pressure reduction, associated with poorer prognosis. In patients with coronary artery disease, inappropriate NDHRI levels, either too high or too low, may lead to inadequate myocardial blood supply or overload, increasing the risk of acute coronary events. Proper management of NDHRI is crucial for maintaining cardiac efficiency and functional status in patients with heart failure, as too low NDHRI might indicate insufficient cardiac output, while too high NDHRI could reflect fluid retention and cardiac overload.

Our findings suggest the potential benefits of maintaining NDHRI within the 40.0%–45.0% range for critically ill patients in the ICU. These associations were consistent across different time frames and medical practices, suggesting potential areas for future investigation. Particularly, the eICU-CRD dataset (17), reflecting more contemporary medical practice patterns from 2014 to 2015, underscores the relevance of our findings to modern clinical practices and enhances their potential significance in the current clinical landscape.

The precise and timely management of critically ill patients in the ICU is crucial (29). Our observations indicate potential benefits of monitoring NDHRI in the ICU setting, though causality cannot be inferred from our data. The dynamic physiological conditions of ICU patients necessitate close monitoring of heart rate patterns to detect any abnormalities promptly. This proactive monitoring is key to initiating diagnostic and therapeutic interventions early, which are essential in managing critical conditions (30). Our research indicates that NDHRI is a valuable predictive tool that helps the medical team foresee disease progression and proactively manage potential complications. Based on these insights, we recommend specific adjustments to patient treatment plans based on NDHRI monitoring outcomes. For example, if NDHRI consistently registers below the 40.0% threshold, it may suggest issues with over-sedation or insufficient metabolic activity, advising adjustments in sedative dosages or increasing physical activity. Conversely, if NDHRI consistently exceeds 45.0%, reevaluation of pain management strategies or adjustment of beta-blocker treatments might be necessary. This strategy of treatment adjustment, based on continuous monitoring of NDHRI, signifies a significant shift towards a more personalized care approach for ICU patients, helping to tailor treatment plans to meet the unique needs of each patient, optimize medication management, and maximize the efficacy of therapeutic interventions while minimizing potential side effects.

Compared to existing literature (31, 32), our study not only corroborates the existing insights on circadian rhythm management and its association with outcomes in ICU patients but also expands on these understandings. We highlight the necessity of further exploring the role of NDHRI in ICU patient management, particularly the mechanisms underlying its adjustment and its potential application in clinical practice.

While our study benefits from a large sample size and the ability to conduct multiple subgroup analyses, it faces certain limitations. Primarily, our dataset originates from the United States, which may limit the generalizability of our findings to ICUs in regions with differing practices and resource availability. Therefore, future research should explore the relationship between NDHRI and patient outcomes across different regions and healthcare systems to enhance the universality and applicability of our findings. International collaboration and multicenter studies are crucial in understanding how differences in culture, medical practices, and resource availability affect the relationship between NDHRI and patient outcomes. Furthermore, despite our rigorous control for multiple confounders, we cannot completely exclude the presence of unaccounted confounding factors. Another limitation is the retrospective design of our study. Future research should consider a prospective design to directly explore the potential causal relationships between adjusting NDHRI and mortality risks. Additionally, further exploration of interventions, such as adjusting NDHRI within an optimal range and its specific impact on patient outcomes, will help clarify the clinical utility of NDHRI adjustments.

Given the feasibility of NDHRI monitoring in clinical practice, especially in resource-limited medical environments, developing and implementing cost-effective, easy-to-use NDHRI monitoring technologies represents another important direction for future research. Advances in technology, such as the development of wearable devices and remote monitoring tools, could further promote the global application of NDHRI, especially in areas where traditional monitoring technologies are less accessible. However, we should cautiously consider the potential of NDHRI as an effective assessment target and maintain an objective perspective on its application prospects. It is essential to recognize the practical value and potential limitations of NDHRI in future medical practices to accurately evaluate and utilize this tool.

In summary, our study underscores the importance of considering circadian rhythms and heart rate indices in the management of ICU patients. Through future clinical trials and research, we look forward to deepening our understanding of how NDHRI impacts the outcomes of critically ill patients and exploring how this knowledge can be translated into practical strategies for improving patient care and outcomes.

## Conclusions

Our extensive analysis of the eICU Collaborative Research Database suggests an association between NDHRI levels of 40.0% and 45.0% and reduced mortality rates among ICU patients. It is crucial to note, however, that these observational findings do not imply causality. Given the U-shaped association observed and the consistent results across various patient subgroups, the relevance of the NDHRI within these specific ranges warrants further investigation through randomized controlled trials.

Future studies must rigorously test NDHRI in diverse ICU settings to establish causality and clinical utility. Randomized trials are crucial for validating and integrating NDHRI into precise, evidence-based patient management, enhancing care for critically ill patients.

## Data Availability

Publicly available datasets were analyzed in this study. This data can be found here: https://physionet.org/content/eicu-crd/get-zip/2.0.
